# Collaborative postgraduate training in family medicine and primary care: Reflections on my visit to South Africa

**DOI:** 10.4102/phcfm.v10i1.1620

**Published:** 2018-04-26

**Authors:** Felicity Goodyear-Smith

**Affiliations:** 1 Department of General Practice and Primary Health Care, University of Auckland, New Zealand

## Abstract

This reflection describes my funded visit to South Africa to assist in primary care research capacity building as Chair, WONCA Working Party on Research (WP-R). The trip included time at the Universities of Walter Sisulu, Limpopo and Stellenbosch to mentor postgraduate students working on master’s and PhD theses. I held one-on-one and group sessions and ran interactive scientific writing workshops. I assisted with the establishment of a Stellenbosch University Family Physician Research Network of faculty academics and family physicians (FP) which will generate research questions from community stakeholders. I also ran a writing workshop at the Joint 5th WONCA Africa and 20th South African National Family Practitioners Conference in Pretoria attended by about 100 conference delegates, ranging from FP registrars to academics with PhDs and peer-reviewed publications. A WP-R panel presentation of international comparisons of primary care systems was also held at this conference, with the countries of Ghana, Malawi, Zimbabwe, Ethiopia and Nigeria presented. During my stay, I reflected on the differences between family medicine in South Africa and in my home country, New Zealand (NZ). In South Africa, there is high prevalence of HIV and tuberculosis, seldom seen in NZ. Donor-funded vertical programmes cause significant fragmentation of care. Family doctors generally work in district hospitals, providing consultancy support to nurse-led clinics. They have a laudable requirement to complete a Master’s in Medicine in conjunction with vocational training. Academic family medicine in South Africa is coming of age. I feel privileged to play a small part in its journey to maturity.

## Introduction

In August 2017, I spent 3 weeks in South Africa. My trip was funded by the National Research Foundation of South Africa, as part of a project on ‘Collaborative Postgraduate Training in Family Medicine and Primary Care’ led by Prof. Bob Mash, Stellenbosch University. The goal of the project was to build research capacity in the discipline of family medicine and primary care, and my role was to mentor researchers at master’s and PhD level, primarily at Walter Sisulu University and the University of Limpopo, and to assist in research capacity building generally.

Strengthening primary health care (PHC) leads to more equitable and cost-effective health care with the overall outcome of a more healthy national population,^1,2^ and this is a South African national health priority.^3,4^ To achieve this, appropriate education of family physicians (FP) as well as basic doctors and clinical associate training is required. Integral to this is strengthening family medicine as an academic discipline, with the ability to conduct relevant primary care research to inform both clinical practice and teaching. Currently, established researchers in the discipline of family medicine have not reached a critical mass.

In this article, I reflect on my experiences during my visit, including the differences between family medicine in South Africa (SA) and in my home country, New Zealand (NZ).

## Family medicine practice in South Africa and New Zealand

The role of the FP in SA differs significantly from vocationally trained FPs (known as general practitioners or GPs) in NZ. The free PHC system in SA is provided through nurse-based clinics and community health centres.^5^ The FPs generally train and work in district hospitals, providing support to the nurse-led clinics in a consultant role.^6^

In NZ, everyone is enrolled with a GP, who works in a community-based clinic providing comprehensive care to patients and families. The practice receives government funding to care for their capitated population, including provision of health prevention activities such as screening. Patients may also pay an out-of-pocket (fee for service) consultation fee, which varies according to the practice and patient demographics (all children aged under 13 years are free, for example). The clinic team includes practice nurses, often a community pharmacist, community workers and occasionally a nurse practitioner. General practitioners do not work in hospitals and generally serve as gatekeepers, referring patients to their specialist colleagues as needed.

South Africa has a high prevalence of HIV. In 2012, it was estimated that 12.2% of the population (approximately 6.4 million people) were living with HIV. The prevalence varies between provinces and between sub-populations. Over a third of women aged 30 to 35 years were estimated to be HIV-positive in 2015.^7^ More than 60% of HIV-positive people are also co-infected with tuberculosis (TB), including resistant and multi-drug resistant strains. In contrast, NZ has a very low prevalence, with an estimated 1153 people living with HIV out of a population of 4.51 million in 2015. The majority (88%) are male, with heterosexual transmission in only about 20%, and over 90% are on antiretroviral therapy (ART).^8^ The prevalence of TB is similarly low, with 302 cases notified in 2014, mostly new disease, as relapsed or reactivation of cases occurs sparingly.^9^ A major risk factor is being born outside of NZ, and cases are more likely to be diagnosed in migrants and refugees. Most NZ GPs are unlikely to see a case of either HIV or TB during their working career, a fact that astonished my South African colleagues, for whom this is an everyday occurrence. This is a health burden that NZ doctors do not have to face.

Although they might be effective in helping get epidemics under control, donor-funded vertical care disease-specific programmes for conditions such as HIV, TB and malaria have a number of negative consequences. They cause significant fragmentation of care, which is becoming more pronounced as HIV-positive people are living longer and developing concurrent non-communicable diseases such as diabetes. These programmes also attract doctors and other health care professionals away from the general and comprehensive delivery of primary care, because the funders can offer greater remuneration. There is an urgent need for an integrated delivery of primary care services.

## Academic family medicine in South Africa

Throughout the world, academic general practice or family medicine, with its own educational content and research, is a relatively young discipline.^10^ It is now recognised as a clinical speciality orientated to personal, family and community-based comprehensive primary care. Studies need to be conducted in the context of community-based settings relevant to the PHC of the population. Relevant research informs both clinical practice and medical teaching. There are now nine departments of family medicine in universities throughout South Africa.^11^ However, it takes time for such departments to mature and for the staff to undertake PhDs and publish papers in peer-reviewed journals. Currently, only two of the nine heads of department hold PhD degrees.

Along with vocational training signed off by the College of Family Physicians of South Africa, there is also now a requirement for family medicine registrars to complete a Master’s in Medicine (MMed) at a university, in line with trainees in other specialities. This includes conducting a research project and submitting a dissertation. This initiative is to be applauded. In contrast, NZ GP registrar training is run by the Royal New Zealand College of General Practitioners with no requirement for a university qualification.

Even if the registrars do not conduct research in the future, having undertaken a dissertation project means they will have a basic understanding of the scientific method. This will serve them well in the future as life-long learners who need to be able to read the published literature and determine its relevance to the context in which they practise.

However, master’s and PhD students need good supervision. In some of the newer departments of family medicine, a critical mass of qualified FPs needs to be reached to provide supervision for the registrars embarking on their research projects. The lack of research supervisory capacity makes the completion of their research projects a limiting step in completing the degree. Once there are academics with PhDs in these departments, they will be able to provide adequate supervision to students at master level.

## Collaborative postgraduate training in family medicine and primary care

The division of family medicine and primary care at Stellenbosch University has set up a collaborative postgraduate training programme to support other departments in more disadvantaged parts of the country. There are now five academics at Stellenbosch with PhDs, who can start supervising PhD candidates at Walter Sisulu University and the University of Limpopo, and hence strengthen research capacity at these two partner institutions. To assist, my role was to travel to Mthatha and Polokwane, and spend time mentoring the postgraduate students at these two universities. I conducted many one-on-one sessions as well as group sessions with some registrars. My approach was to examine their research questions, ensure that their aims and objectives matched up with these and then to explore with them how they were going to answer this question (determine the methodology).

I also ran a number of interactive writing workshops. Firstly, I addressed the structure of scientific writing, which follows the same principles regardless of whether the work is a research proposal, ethics application, report, dissertation, thesis or scientific paper. Secondly, I gave a number of writing tips, of particular value when English is not the first language, and when needed, I also gave a presentation on writing for publication. Participants worked in small groups to identify the errors in a badly written abstract and to rewrite it addressing grammar, spelling, syntax, punctuation and other issues. I ran a writing workshop at the Joint 5th WONCA Africa and 20th SA National Family Practitioners Conference in Pretoria attended by about 100 conference delegates, ranging from FP registrars to academics with PhDs and peer-reviewed publications. I left my presentations and other materials with the organisers wherever I went, for them to reuse and disseminate as they wished.

## Practice-based research network

I also participated in a workshop on developing a local practice-based research network (PBRN) led by Prof. Bob Mash and attended by faculty academics and FPs. I gave a presentation on international perspectives on PBRNs in primary care, which was followed by discussion on how to set up a network that would work in a South African setting. The key component of a PBRN is a group of practices principally devoted to the care of patients which act as a ‘laboratory’ to study populations of patients and care providers in community-based settings. The research questions are generated from community stakeholders, not university researchers, and the study is conducted with, not on, the participants. At the end of the meeting, they had developed a framework document for the Stellenbosch University Family Physician Research Network (SUFPREN) and had put in place plans to progress this initiative.

## Comparative primary care panels

Over the past 5 years, through the WONCA Working Party on Research (WP-R), of which I am currently the Chair, I have arranged a series of international comparisons of primary care systems with panel presentations at regional and world conferences.^12,13,14,15,16^ These presentations use standardised templates to enable system comparisons, have mostly focused on health systems of developing nations and have consistently flagged the importance of universal health care coverage and primary care contribution to the health system. Prof. Bob Mash led a similar panel workshop at the WONCA Africa conference, with Drs Osa Olayem, Martha Makwero, Sundanda Ray, Meseret Zerihun and Abraham Gyuse presenting the primary care systems in their countries of Ghana, Malawi, Zimbabwe, Ethiopia and Nigeria, respectively. This will also lead to a peer-reviewed publication, currently under submission.

## Conclusion

In conclusion, academic family medicine and PHC in South Africa is coming of age. I feel privileged to have been able to play a small part in its journey to maturity. I am grateful to Prof. Bob Mash and to the National Research Foundation for this rewarding opportunity.
